# Long noncoding RNA antisense noncoding RNA in the INK4 locus inhibition alleviates airway remodeling in asthma through the regulation of the microRNA‐7‐5p/early growth response factor 3 axis

**DOI:** 10.1002/iid3.823

**Published:** 2023-04-12

**Authors:** Liyan Wang, Xueru Liu

**Affiliations:** ^1^ Department of Pediatrics Wuhan Third Hospital Wuhan China; ^2^ Department of Respiratory Medicine, Wuhan Children's Hospital, Tongji Medical College Huazhong University of Science & Technology Wuhan China

**Keywords:** airway remodeling, asthma, lncRNA ANRIL, migration, miR‐7‐5p, proliferation

## Abstract

Asthma, a chronic inflammatory disease of the airways, clinically manifests as airway remodeling. The purpose of this study was to probe the potential role of long noncoding RNA (lncRNA) antisense noncoding RNA in the INK4 locus (lncRNA ANRIL) in the proliferation and migration of airway smooth muscle cell (ASMC) and to explore its potential mechanisms in asthma. Serum samples were obtained from 30 healthy volunteers and 30 patients with asthma. Additionally, platelet‐derived growth factor‐BB (PDGF‐BB) was used to induce airway remodeling in ASMCs. The level of lncRNA ANRIL and microRNA (miR)‐7‐5p in serum samples were measured by quantitative reverse transcriptase polymerase chain reaction (qRT‐PCR). TargetScan predicted the binding site of miR‐7‐5p to early growth response factor 3 (EGR3) and validated the results using a dual‐luciferase reporter assay. 3‐(4,5‐dimethylthiazol‐2‐yl)‐2,5‐diphenyl‐2H‐tetrazolium bromide (MTT) and Transwell assays were used to detect cellular proliferation and migration, respectively. Subsequently, changes in proliferation‐ and migration‐related genes were verified using western blot analysis and qRT‐PCR. These results indicate that lncRNA ANRIL was upregulated in the serum and PDGF‐BB‐induced ASMCs of patients with asthma, whereas miR‐7‐5p expression was reduced. EGR3 was a direct target of miR‐7‐5p. LncRNA ANRIL silencing inhibited the proliferation or migration of ASMCs induced by PDGF‐BB through miR‐7‐5p upregulation. Mechanistic studies indicated that miR‐7‐5p inhibits the proliferation or migration of PDGF‐BB‐induced ASMCs by decreasing EGR3 expression. EGR3 upregulation reverses the role of miR‐7‐5p in airway remodeling. Thus, downregulation of lncRNA ANRIL inhibits airway remodeling through inhibiting the proliferation and migration of PDGF‐BB‐induced ASMCs by regulating miR‐7‐5p/EGR3 signaling.

## Graphical abstract

In this study, we investigated the function of long noncoding RNAs (lncRNA) ANRIL in proliferation and migration of airway smooth muscle cells (ASMCs), and to explore the role and potential mechanisms in asthma. The data indicated that The downregulation of lncRNA ANRIL inhibits airway remodeling through regulating miR‐7‐5p/EGR3 signaling to inhibit proliferation and migration of platelet‐derived growth factor‐BB‐induced ASMCs.

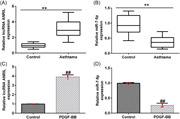

## 1 INTRODUCTION

Asthma is a chronic inflammatory disease of the airway, with a worldwide prevalence of approximately 3.8/1000 and a high prevalence in all age groups.^1^, ^2^ In many countries, especially children, the prevalence of asthma has increased, and the differential diagnosis of asthma varies by age.^3^ Research has shown that asthma has several complex causative factors, including genetics and respiratory viral infections.^4^ Clinically, asthma presents with symptoms such as breathlessness, chest tightness, and coughing, and in severe cases, can lead to respiratory failure, lung infection, or sudden death.^5^ Currently, the main treatment modalities for asthma are anti‐inflammatory agents and bronchodilators. For example, asthma may be relieved by taking leukotriene receptor antagonists.^6^ However, traditional drugs have many side effects, and research on new treatments and biomarkers is key to the treatment of asthma.

As one of the main pathological characteristics of asthma, airway remodeling manifests as damage of bronchial epithelium, smooth muscle cell hyperplasia, proliferation of subepithelial fibroblasts activation, and increased angiogenesis.^7^, ^8^, ^9^, ^10^, ^11^ The main cellular components of the airway are the airway epithelial cells and the airway smooth muscle cells (ASMCs).^12^ Studies have shown that one of the main reasons for airway remodeling in patients with asthma is an increase in the proliferation and migration of ASMCs.^13^, ^14^ Several inflammatory mediators are essential for airway remodeling.^15^ The asthma‐associated cytokine interleukin (IL)‐4 and its counterpart interferon (IFN)‐g were shown to orchestrate an epithelial polarization in the airways.^16^ Further, not only pro‐inflammatory mediators are crucial but also anti‐inflammatory mediators such as secretoglobin 1A1 and IL‐37.^17^, ^18^, ^19^ The suppressive secretoglobin 1A1 in cells of the lower airways following allergen immunotherapy,^17^ while IL‐37 was shown to regulate allergic inflammation by counterbalancing pro‐inflammatory IL‐1 and IL‐33, a known contributor of airway remodeling in asthma airway disease.^18^, ^19^ Clinical evidence also suggests that PDGF‐BB is upregulated in patients with asthma and that PDGF‐BB induces ASMCs proliferation and migration, ultimately leading to increased airway remodeling.^20^, ^21^ These results imply that inhibition of ASMC migration and proliferation may be an effective therapeutic method for asthma.

Long noncoding RNAs (lncRNAs) are RNA molecules more than 200 nucleotides in length.^22^, ^23^ Numerous studies have found that lncRNAs are involved in various biological activities and are tissue‐specific, suggesting that they could be potential diagnostic markers for disease.^24^ Recent research has revealed that lncRNAs play a key role in airway remodeling and regulation of the proliferation and migration of ASMCs.^12^, ^21^ For example, lncRNA brain cytoplasmic RNA 1 (BCYRN1) promotes ASMCs proliferation and migration through the upregulation of receptor potential 1.^25^ LncRNA antisense noncoding RNAs at the INK4 locus (ANRIL) have been reported to play a significant role in cancers and cardiovascular diseases.^26^ Silencing them inhibits proliferation and migration in liver cancer.^27^ Furthermore, they affect coronary artery disease through the miR‐181b and NF‐κB signaling pathways.^28^

MicroRNAs (miRNAs) are small noncoding RNA that regulate messenger RNA (mRNA) stability and protein translation with specific sequences in the 3′untranslated regions (3′UTRs) of mRNA.^29^, ^30^ MiRNAs were shown to be of relevance in the setting of asthma and associated lung inflammation, such as miR‐3935 in sputum was shown to target Prostaglandin EP3 receptor as selective target in allergen‐specific immunotherapy.^31^, ^32^ The lncRNA ANRIL targets miR‐7‐5p binding to participate in disease regulation. ANRIL was found to play a protective role in hypoxia‐induced injury in H9C2 cells by regulation of the miR‐7‐5p and sirtuin1 (SIRT1) axis.^33^ Additionally, the lncRNA ANRIL regulates the malignant phenotype of T‐cell acute lymphocytic leukemia (T‐ALL) cells by regulating the miR‐7‐5p and transcription factor 4 (TCF4) axis.^34^ Recently, it was found that lncRNA ANRIL could promote inflamed periodontal ligament stem cells osteogenic differentiation through modulation of the miR‐7‐5p and insulin‐like growth factor type 1 receptor (IGF‐1R) pathways.^35^ However, whether lncRNA ANRIL could affect airway remodeling in asthma by regulating miR‐7‐5p remains unclear.

In the current study, we hypothesized that lncRNA ANRIL is involved in airway remodeling in asthma by regulating miR‐7‐5p expression. Therefore, we studied the role of the lncRNA ANRIL in the proliferation and migration of ASMCs in this research and analyzed its underlying molecular mechanisms.

## 2 MATERIALS AND METHODS

### 2.1 Participants and ethics statement

For this study, 30 patients with asthma and 30 healthy volunteers were recruited at Wuhan Third Hospital. Inclusion criteria: (1) patients diagnosed with bronchial asthma according to GINA guidelines (2016); (2) no history of bronchial asthma or other allergic diseases; (3) patients over 18 years old; (4) no inflammatory diseases, hematological malignancies or tumors; (5) 4 weeks without infection. Exclusion criteria: (1). Patients with history of bronchial asthma or other allergic diseases; (2) patients <18 years old; (3) patients have been treated with corticosteroids, immunosuppressants, immunomodulators, or inflammatory mediator antagonists for 4 weeks; (4) Infection within 4 week; patients with inflammatory disease, hematological malignancy, or tumor. The characteristics of asthmatic patients were presented in Table 1. Blood samples were collected from participants with EDTA‐coagulation vessels and centrifuged at 1000 g for 10 min to separate the serum. Each participant signed an informed consent form approved by the doctrine committee. This study was approved by the Ethics Committee of Wuhan Third Hospital (Approval number: KY2022‐034).

**Table 1:** The characteristics of asthmatic patients and healthy controls.

Characteristics	Asthmatic patients (*n* = 30)	Healthy controls (*n* = 30)	*p*
Age (years)	26–34	25–35	‐
Gender (male/female)	16/14	12/18	>.05
FEV1 (%)	68.23 ± 2.11	100.51 ± 3.17	<.01
Serum total IgE (IU/mL)	174.02 ± 12.32	26.45 ± 9.13	<.01

Abbreviation: FEV1, forced expiratory volume in 1 s.

### 2.2 Cell culture

Human airway smooth muscle cells (HASMCs) were supported by American Type Culture Collection. The cells were grown in a six‐well plate in dulbecco's modified eagle medium (DMEM; Gibco; Thermo Fisher Scientific, Inc.) with 10% fetal bovine serum (Gibco; Thermo Fisher Scientific, Inc.) and 1% penicillin‐streptomycin at 37°C with 5% CO_2_. Cells at passages 2–3 were used for following experiments.

For the PDGF‐BB induction group, HASMCs were first serum deprived for 24 h and then induced with 25 ng/mL PDGF‐BB (R&D Systems) for 24 h.^36^ Experiments were repeated three times independently.

### 2.3 Western blot assay

Protein levels were detected by western blot assay.^37^ For total protein extraction, radioimmunoprecipitation assay lysis buffer was applied. The supernatants were collected by centrifugation at 12,000 g for 10 min and assayed for total protein using a bicinchoninic acid kit (Beyotime). The samples were processed using sodium dodecyl sulfate‐polyacrylamide gel electrophoresis (SDS‐PAGE), and the membrane transfer procedure was conducted on a polyvinylidene fluoride membrane (Millipore). Subsequently, the membranes were blocked by 5% milk for 1 h. The membrane was then incubated overnight at 4°C with primary antibodies against PCNA (cat. no. #13110; 1:1000; Cell Signaling Technology, Inc.), MMP9 (cat. no. AF5228; 1:500; Affbiotech), early growth response factor (EGR3; cat. no. #2559; 1:1000; Cell Signaling Technology, Inc.), and GAPDH (cat. no. ab181602; 1:10000; Abcam). The next day, the membrane was washed five times with tris‐buffered saline Tween‐20  buffer. The membranes were then incubated with horseradish peroxidase (HRP)‐labeled secondary antibody (cat. no. AS1107; 1:10000; ASPEN). After 2 h, the bands were visualized with an electrogenerated chemiluminescence (ECL) luminescent solution (Beyotime Institute of Biotechnology). Experiments were repeated for three times.

### 2.4 Quantitative reverse transcriptase polymerase chain reaction (qRT‐PCR)

Total RNA was collected from serum samples and HASMCs (10^6^ cells per well in six‐well plates) using RNA‐easy Isolation Reagent (Vazyme), and complementary DNA (cDNA) was obtained with a reverse transcription kit (Takara). cDNA was quantified by RT‐PCR using SYBR Green (Takara). GAPDH for mRNA and U6 for miRNA were used as the internal controls. The primers were synthesized by Sangon Biotech, and the sequences are presented in Table 2. Relative quantification of genes was detected using the 2^‐ΔΔCt^ method.^38^ Experiments were performed at least for three times.

**Table 2:** Primer sequences for PCR.

Gene	Forward primer (5′‐3′)	Reverse primer (5′‐3′)
ANRIL	TGCTCTATCCGCCAATCAGG	GGGCCTCAGTGGCACATACC
miR‐7‐5p	AAAACTGCTGCCAAAACCAC	GCTGCATTTTACAGCGACCAA
PCNA	CTAGCCATGGGCGTGAAC	GAATACTAGTGCTAAGGTGTCTGCAT
MMP9	TTCGCGTGGATAAGGAGTTC	CCTCC ACTCCTTCCCAGTCT
EGR3	GACATCGGTCTGACCAACGAG	GGCGAACTTTCCCAAGTAGGT
GAPDH	CGGAGTCAACGGATTTGGTCGTAT	AGCCTTCTCCATGGTGGTGAAGAC
U6	CTCGCTTCGGCAGCACATATACT	ACGCTTCACGAATTTGCGTGTC

### 2.5 Cell transfection

ANRIL siRNA, control siRNA, miR‐7‐5p mimic, miR‐7‐5p inhibitor, EGR3 overexpression plasmid (EGR3‐plasmid), and control plasmid were constructed by GenePharma. The siRNAs or plasmids were transfected into HASMCs (5 × 10^4^ cells per well in 24‐well plates) using Lipofectamine 2000 (Life Technologies) as per the manufacturer's instructions. After 48 h, the transfection efficiency was measured by qRT‐PCR. Experiments were performed at least for three times.

### 2.6 MTT assay for proliferation

To measure cell proliferation, an MTT assay was conducted, as described previously.^39^ Cells (3 × 10^4^ cells/well) were seeded in a 96‐well plate and cultivated for 48 h before the MTT assay. MTT (Beyotime) (20 µL) was added to each well and the cells were cultured for 4 h at 37°C. Finally, a plate reading spectrophotometer (BioTek Instruments, Inc.) was used to measure the absorbance at 570 nm. Experiments were independently repeated three times.

### 2.7 Transwell assay for migration

Cell migration was assessed by the Transwell assay.^36^ Briefly, serum‐free and 10% serum‐containing cultures were separately added to the upper and lower layers of the 24‐well plate with an 8 μm pore size, and cells were incubated in the upper layer for 24 h. Cells that penetrated the lower layer were fixed with 4% methanol (Sangon Biotech) and stained with crystal violet (Sangon Biotech). The cells were quantified using an inverted microscope (Olympus). Experiments were independently repeated three times.

### 2.8 Bioinformatics and dual‐luciferase reporter assay

Potential targets of miR‐7‐5p were predicted using TargetScan (http://www.targetscan.org). As previously described, a dual‐luciferase reporter assay was conducted to verify interactions.^40^ EGR3‐WT or EGR3‐MUT were cotransfected with miR‐7‐5p mimic into HASMCs (5 × 10^4^ cells per well in 24‐well plates) with transfection reagent. After 48 h, transfection efficiency was assessed using a dual luciferase assay kit (Beyotime). Experiments were independently repeated for three times.

### 2.9 Statistical analysis

SPSS analysis (version 20.0; IBM Corp.) was performed as the statistical analysis, and all data were expressed as mean ± standard deviation. We used the Kolmogorov–Smirnov test to determine the normality of the data in SPSS. Statistical comparisons among multiple groups were analyzed by one‐way analysis of variance followed by a Tukey's post‐hoc test, and Student's *t*‐test was conducted to analyze the statistical comparisons among two groups. *p* < .05 means the difference is significant.

## 3 RESULTS

### 3.1 Expression of lncRNA ANRIL and miR‐7‐5p in serum of patients with asthma and PDGF‐BB‐induced HASMCs

**Figure 1:**
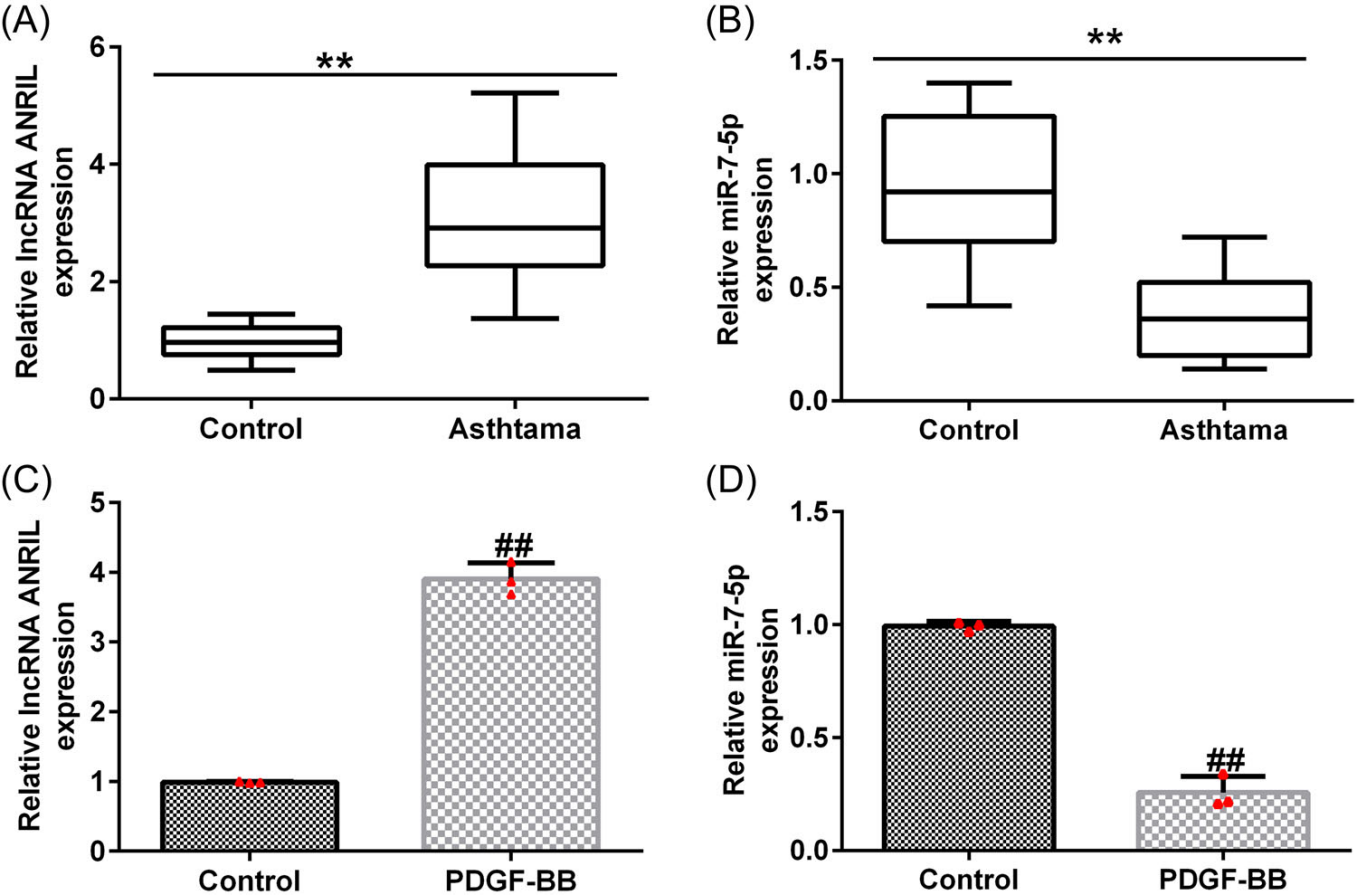
Expression of long noncoding RNAs (lncRNA) ANRIL and miR‐7‐5p in serum of patients with asthma and platelet‐derived growth factor‐BB (PDGF‐BB)‐induced human airway smooth muscle cells (HASMCs). (A, B) The quantitative reverse transcriptase polymerase chain reaction (qRT‐PCR) is used to detect the expression of lncRNA ANRIL and miR‐7‐5p in serum of patients with asthma (*n* = 30). **p < .01; (C, D) The qRT‐PCR is used to detect the expression of lncRNA ANRIL and miR‐7‐5p in PDGF‐BB‐induced HASMCs (*n* = 3). ##*p* < .01 versus control group.

To examine the potential role of lncRNA ANRIL and miR‐7‐5p in asthma, we collected serum samples from 30 patients with asthma and 30 healthy volunteers. qRT‐PCR results indicated that lncRNA ANRIL was significantly enhanced, while miR‐7‐5p was decreased in the serum of asthmatic cohorts compared to the healthy group (Figure 1A,B). Additionally, PDGF‐BB‐induced ASMCs proliferation and migration are involved in asthma pathogenesis.^13^ We cultured HASMCs with PDGF‐BB (25 ng/mL) for 24 h and examined the expression of lncRNA ANRIL and miR‐7‐5p by qRT‐PCR. In line with the results in patients with asthma, lncRNA ANRIL was notably increased whereas miR‐7‐5p was downregulated in PDGF‐BB‐induced HASMCs (Figure 1C,D). These results revealed that lncRNA ANRIL and miR‐7‐5p are involved in asthma and can be used as markers to predict asthma.

### 3.2 LncRNA ANRIL negatively regulates miR‐7‐5p in HASMCs

**Figure 2:**
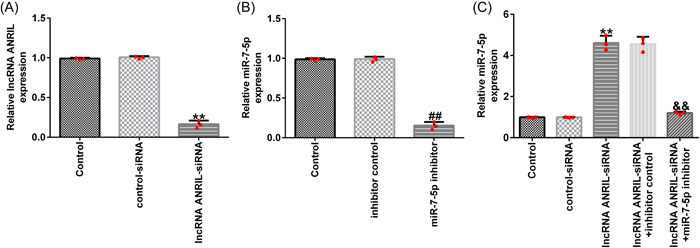
Long noncoding RNAs (lncRNA) ANRIL negatively interacted with miR‐7‐5p in human airway smooth muscle cells. (A) The expression of lncRNA ANRIL at transfected cells is measured through quantitative reverse transcriptase polymerase chain reaction (qRT‐PCR); (B) The expression of miR‐7‐5p in the transfected cells is detected by qRT‐PCR; (C) The level of miR‐7‐5p is measured by qRT‐PCR after transfection for 48 h. *n* = 3; **p < .01 versus control‐siRNA; ##*p* < .01 versus inhibitor control; &&*p* < .01 versus lncRNA ANRIL‐siRNA+inhibitor control.

The relationship between lncRNA ANRIL and miR‐7‐5p in HASMCs was determined. The results showed that lncRNA ANRIL‐siRNA notably suppressed the expression of lncRNA ANRIL in HASMCs compared to that in the control siRNA group (Figure 2A). Similarly, the miR‐7‐5p inhibitor significantly reduced the level of miR‐7‐5p in HASMCs compared to the inhibitor control group (Figure 2B). Additionally, lncRNA ANRIL‐siRNA profoundly increased miR‐7‐5p expression in HASMCs compared to the control siRNA group, and this result was significantly reversed by the miR‐7‐5p inhibitor (Figure 2C). These data suggest that the lncRNA ANRIL negatively regulated miR‐7‐5p expression in HASMCs.

### 3.3 LncRNA ANRIL‐siRNAs inhibits PDGF‐BB‐induced ASMCs proliferating and migrating through increasing miR‐7‐5p expression

**Figure 3:**
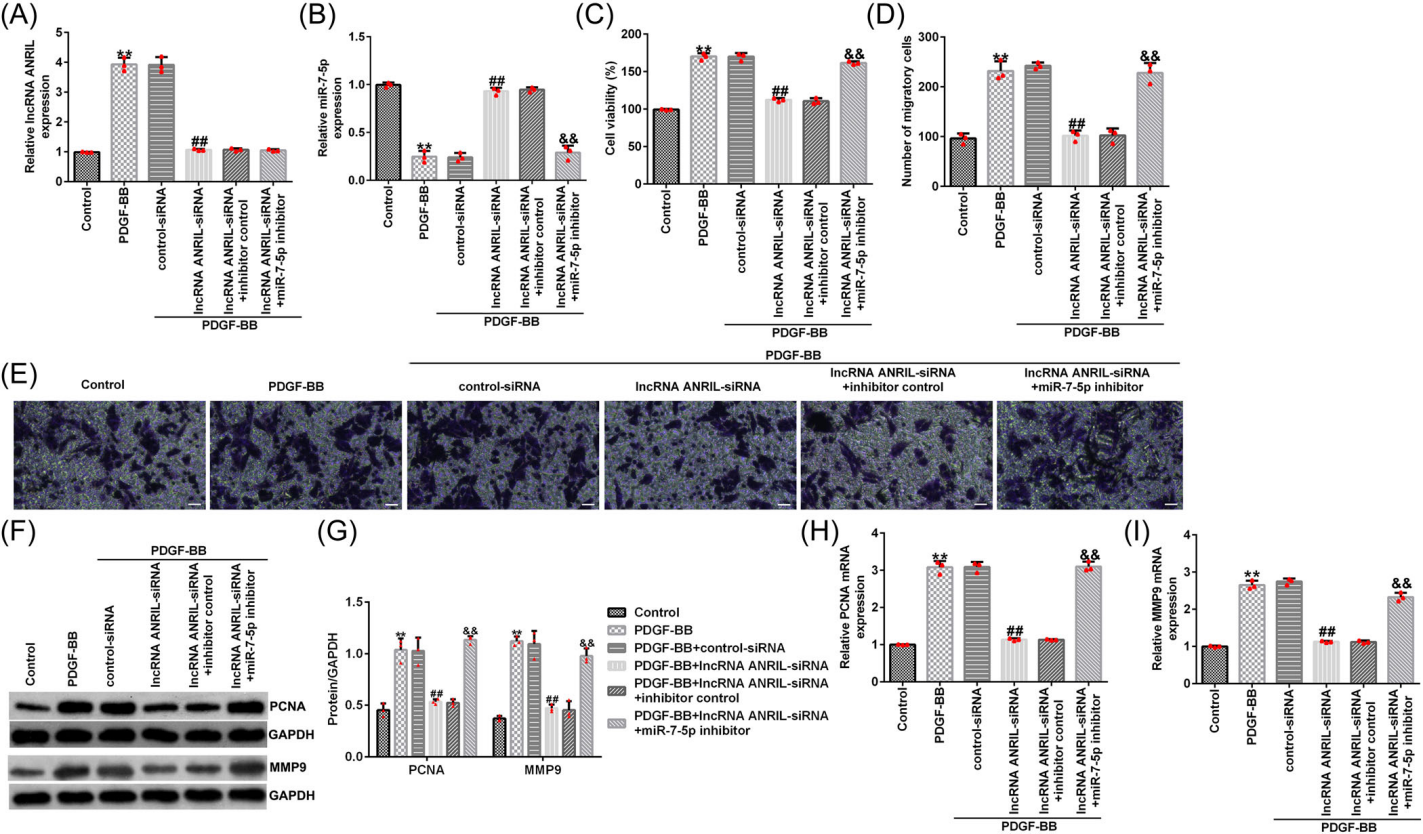
Silencing of long noncoding RNAs (LncRNA) ANRIL inhibited proliferation and migration in platelet‐derived growth factor‐BB (PDGF‐BB)‐induced ASMCs through upregulating miR‐7‐5p. (A, B) The quantitative reverse transcriptase polymerase chain reaction (qRT‐PCR) is performed to measure the expression of lncRNA ANRIL and miR‐7‐5p in transfected cells; (C) Proliferation is tested by the MTT assay; (D, E) Migration is tested by the Transwell assay (bar =  50 μm); (F) western blot analysis is used to evaluate the expression levels of PCNA and MMP9; (G) PCNA/GAPDH and MMP9/GAPDH were determined; (H, I) qRT‐qPCR is used to evaluate the mRNA expression levels of PCNA and MMP9. *n* = 3; **p < .01 versus Control; ##*p* < .01 versus PDGF‐BB + control‐siRNA; &&*p* < .01 versus PDGF‐BB + clncRNA ANRIL‐siRNA+inhibitor control.

Then we investigated whether miR‐7‐5p is involved in the function of lncRNA ANRIL in asthma, we performed a loss‐of‐function experiment. The results indicated that the level of lncRNA ANRIL was notably higher and miR‐7‐5p was significantly lower in the PDGF‐BB group than in the control group. Compared to the control siRNA, lncRNA ANRIL was significantly decreased in the PDGF‐BB‐treated lncRNA ANRIL‐siRNA group; nevertheless, the level of miR‐7‐5p was significantly enhanced. Furthermore, lncRNA ANRIL co‐transfected with the miR‐7‐5p‐inhibitor reversed these results (Figure 3A,B).

We then examined cell proliferation by MTT and the findings showed that HASMCs proliferation was remarkably promoted in the PDGF‐BB group compared to that in the control group (Figure 3C). However, compared to the PDGF‐BB + control‐siRNA group, lncRNA ANRIL‐siRNA significantly inhibited HASMCs proliferation, whereas this inhibition was reversed by miR‐7‐5p inhibitor (Figure 3C). The results of the Transwell assay showed that HASMCs migrated significantly faster in the PDGF‐BB group than in the control group (Figure 3D,E). However, the enhanced cell migration induced by PDGF‐BB was significantly reduced by lncRNA ANRIL‐siRNA, whereas miR‐7‐5p‐inhibitor inhibited this reduction (Figure 3D,E). PCNA is an important regulatory gene for cell proliferation, and MMP9 is closely related to cell migration, so PCNA and MMP9 were detected in this study to further confirm the effect of lncRNA ANRIL/miR‐7‐5p on the proliferation and migration of HASMCs. Furthermore, western blot analysis and qRT‐PCR showed enhanced expression of PCNA and MMP9 in PDGF‐BB‐treated HASMCs (Figure 3F–I). However, compared with the PDGF‐BB + control‐siRNA group, lncRNA ANRIL‐siRNA significantly inhibited the expression of PCNA and MMP9 in the lncRNA ANRIL‐siRNA group. Cotransfection of lncRNA ANRIL‐siRNA with miR‐7‐5p‐inhibitor significantly enhanced the expression of PCNA and MMP9 (Figure 3F–I). These results revealed that lncRNA ANRIL‐siRNA inhibited PDGF‐BB‐induced proliferation and migration of HASMCs through the upregulation of miR‐7‐5p.

### 3.4 EGR3 was a direct target of miR‐7‐5p

**Figure 4:**
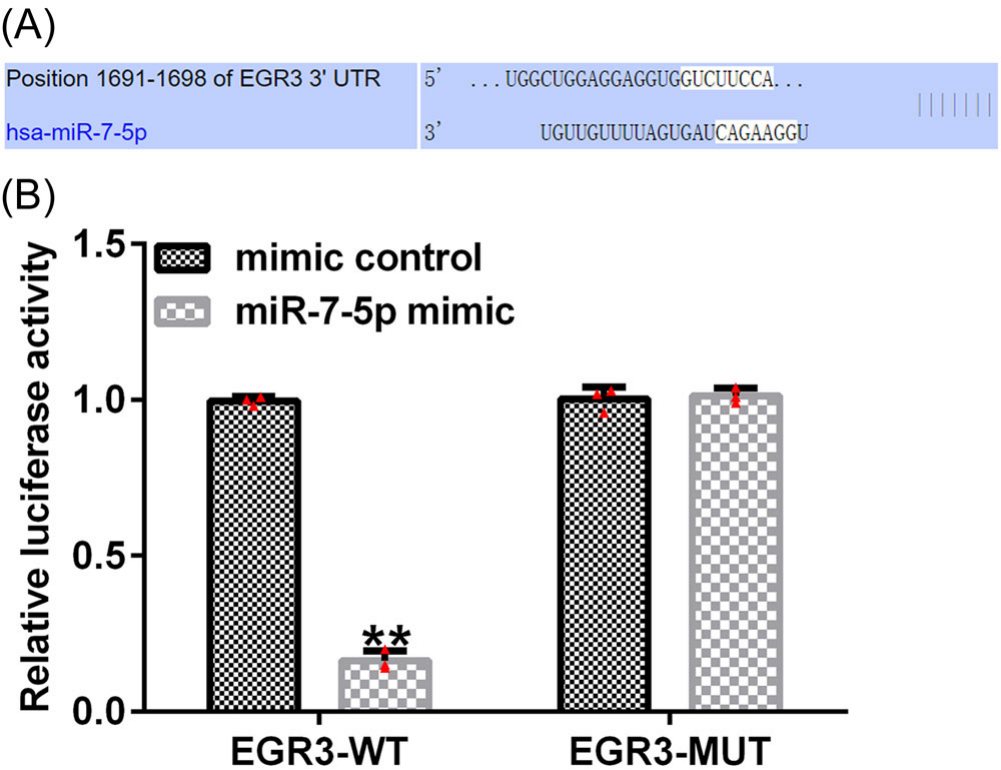
EGR3 directly targets miR‐7‐5p. (A) TargetScan is used to analyze the binding sites of miR‐7‐5p; (B) Dual‐Luciferase Reporter Assay is used to confirm the binding sites between EGR3 with miR‐7‐5p. *n* = 3; **p < .01 versus mimic control.

To explore the mechanisms of miR‐7‐5p in the proliferation and migration of PDGF‐BB‐induced HASMCs, we used the TargetScan database to predict the potential targets of miR‐7‐5p. These results indicated that miR‐7‐5p has binding sites for EGR3 (Figure 4A). Additionally, the dual‐luciferase reporter assay revealed that miR‐7‐5p mimic specifically decreased the luciferase activity of EGR3‐WT; but not the luciferase activity of EGR3‐MUT (Figure 4B). This result further confirmed that EGR3 was the binding target of miR‐7‐5p.

### 3.5 EGR3 is upregulated in the serum of patients with asthma and PDGF‐BB‐induced HASMCs

**Figure 5:**
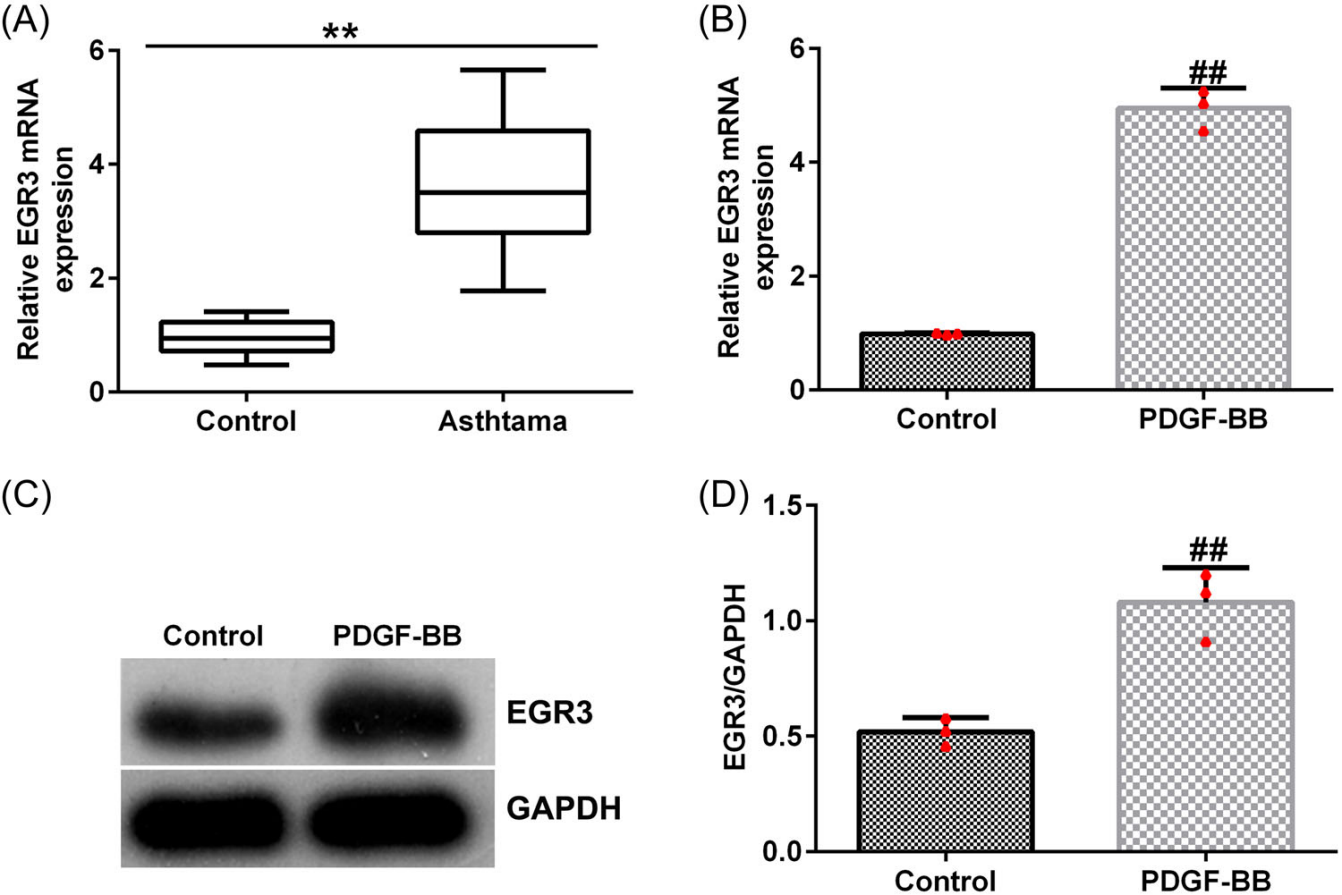
Expression of EGR3 in serum of patients with asthma and platelet‐derived growth factor‐BB (PDGF‐BB)‐induced human airway smooth muscle cells (HASMCs). (A) Quantitative reverse transcriptase polymerase chain reaction (qRT‐PCR) is used to measure the expression of EGR3 in serum of patients with asthma (*n* = 30; **p < .01); (B) qRT‐qPCR is used to measure the expression of EGR3 in PDGF‐BB‐induced HASMCs (*n* = 3). (C) Western blot analysis is performed to detect the expression of EGR3 in PDGF‐BB‐induced HASMCs (*n* = 3). (D) EGR3/GAPDH was calculated (*n* = 3). ##*p* < .01 versus control.

To measure the mRNA levels of EGR3 in the serum of 30 patients with asthma and 30 healthy volunteers, qRT‐PCR was used. The results displayed that the expression of EGR3 was prominently higher in patients with asthma than in healthy volunteers (Figure 5A). Similarly, the mRNA and protein expression of EGR3 was higher in PDGF‐BB‐treated HASMCs than in the control group (Figure 5B–D). These results verified that EGR3 expression is elevated in asthma.

### 3.6 MiR‐7‐5p negatively regulates EGR3 in HASMCs

**Figure 6:**
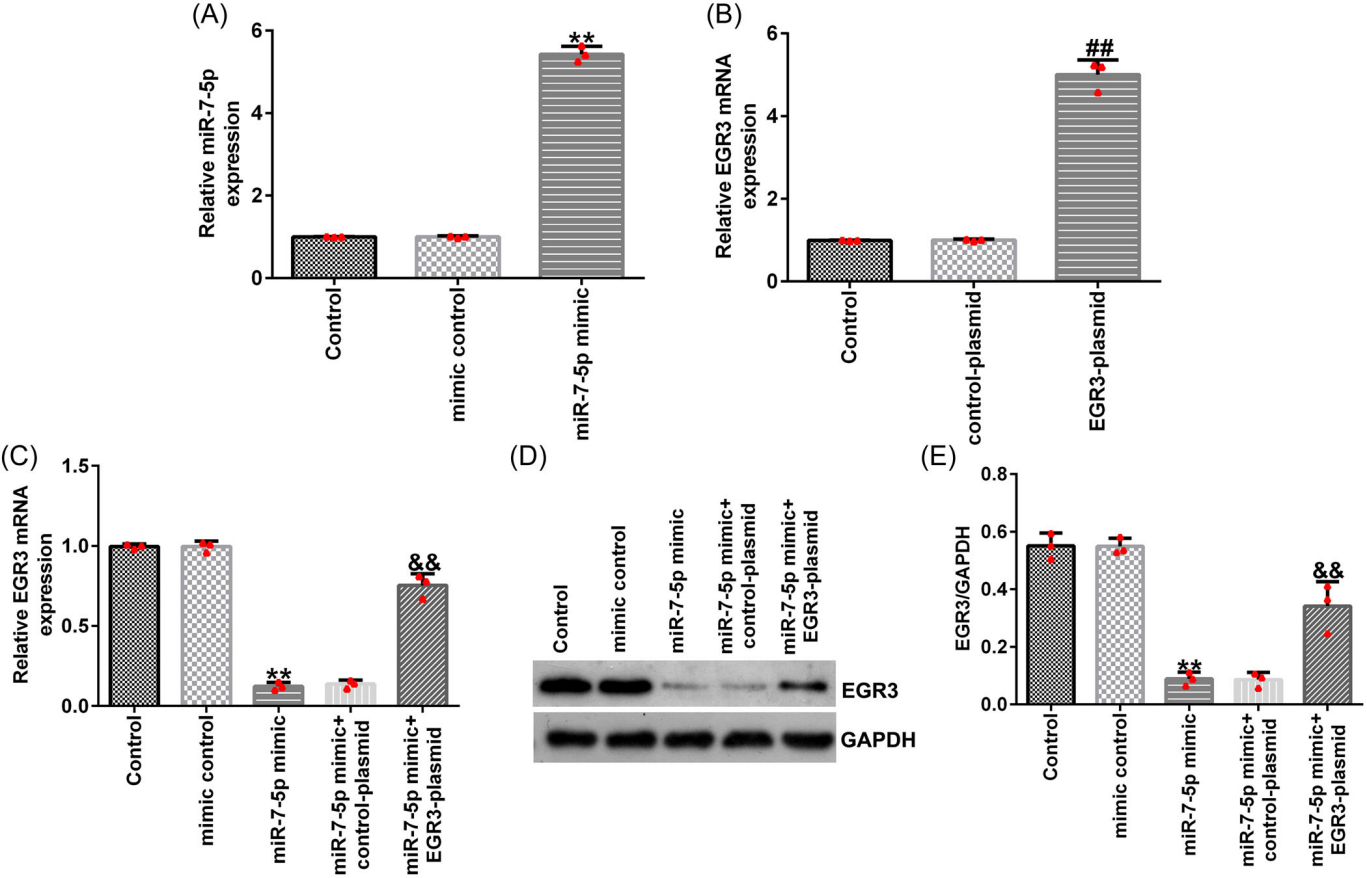
MiR‐7‐5p negatively regulated with EGR3 in human airway smooth muscle cells. (A) The expression of miR‐7‐5p in transfected cells is detected using quantitative reverse transcriptase polymerase chain reaction (qRT‐PCR); (B–D) The expression of EGR3 in the transfected cells is detected using qRT‐PCR and western blot analysis assay. (E) EGR3/GAPDH was calculated. *n* = 3; **p < .01 versus mimic control; ##*p* < .01 versus control‐plasmid; &&*p* < .01 versus miR‐7‐5p mimic+control‐plasmid.

The relationship between miR‐7‐5p and EGR3 in HASMCs was also determined in this study. The results showed that the miR‐7‐5p mimics profoundly enhanced the level of miR‐7‐5p in HASMCs (Figure 6A). EGR3‐plasmid enhanced the expression of EGR3 in ASMCs compared to the control plasmid group (Figure 6B). Additionally, compared to the mimic‐control group, the expression level of EGR3 in HASMCs was significantly downregulated by the miR‐7‐5p mimic. However, miR‐7‐5p mimics co‐expression with the EGR3‐plasmid reversed this result (Figure 6C–E). These data imply that miR‐7‐5p negatively regulated EGR3 expression in HASMCs.

### 3.7 MiR‐7‐5p mimic inhibits proliferation and migration of PDGF‐BB‐induced HASMCs via EGR3

**Figure 7:**
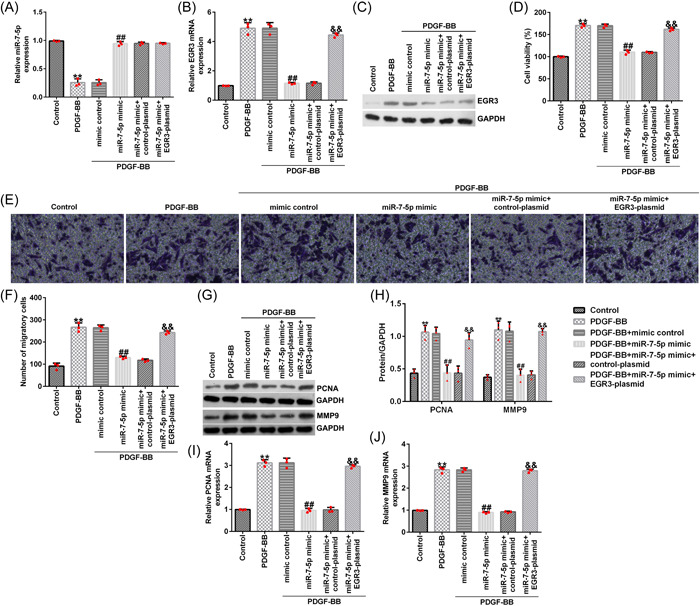
MiR‐7‐5p mimic inhibited proliferation and migration in platelet‐derived growth factor‐BB (PDGF‐BB)‐induced human airway smooth muscle cells (HASMCs) via EGR3. (A) Quantitative reverse transcriptase polymerase chain reaction (qRT‐PCR) is used to measure the expression of miR‐7‐5p in transfected cells; (B, C) qRT‐qPCR and western blot analysis assay are used to measure the expression of EGR3 in transfected cells; (D) Proliferation is tested with MTT assay; (E, F) Migration is tested by the Transwell assay (bar = 50 μm); (G) western blot analysis is used to evaluate the expression levels of PCNA and MMP9; (H) PCNA/GAPDH and MMP9/GAPDH were determined; (I, J) qRT‐qPCR is used to evaluate the expression levels of PCNA and MMP9. *n* = 3; **p < .01 versus control; ##*p* < .01 versus PDGF‐BB + mimic control; &&*p* < .01 versus PDGF‐BB + miR‐7‐5p mimic+control‐plasmid.

Finally, we further elucidated the effects and mechanism of miR‐7‐5p on the proliferation and migration of PDGF‐BB‐induced HASMCs. Findings indicated that PDGF‐BB significantly reduced miR‐7‐5p expression (Figure 7A) and enhanced the mRNA and protein expression of EGR3 (Figure 7B,C). However, the miR‐7‐5p mimic significantly increased miR‐7‐5p and significantly decreased EGR3 mRNA and protein expression after PDGF‐BB induction (Figure 7B,C). After the miR‐7‐5p mimic and EGR3‐plasmid were co‐transfected, the reduced expression of EGR3 was profoundly increased. (Figure 7B,C).

Based on this, we detected cell proliferation using an MTT assay. The results showed that the proliferation of PDGF‐BB‐treated HASMCs was faster than that of the control group (Figure 7D). The miR‐7‐5p mimics significantly reduced cell proliferation (Figure 7D), and this effect was reversed by EGR3‐plasmid (Figure 7D). Subsequently, we detected the effects of miR‐7‐5p and EGR3 on cellular migration using a Transwell assay. The results demonstrated a significantly higher migration of HASMCs induced by PDGF‐BB compared to the control group, while miR‐7‐5p mimics reduced migration (Figure 7E,F). The potential role of miR‐7‐5p in HASMCs migration was reversed after transfection with EGR3‐plasmid (Figure 7E,F). Moreover, the enhanced protein and mRNA expression of PCNA and MMP9 in PDGF‐BB‐treated HASMCs was significantly reduced by the miR‐7‐5p mimic (Figure 7G–J), and these decreases were significantly reversed by EGR3‐plasmid (Figure 7G–J). These findings suggest that the miR‐7‐5p mimic prevents the proliferation and migration of PDGF‐BB‐induced HASMCs by reducing EGR3 expression.

## 4 DISCUSSION

Asthma is the most widespread chronic airway disease in children and adults and presents with various symptoms such as coughing and breathlessness.^1^, ^5^, ^41^ Asthma is a complex disease that is generally caused by genetic and environmental factors. Currently, anti‐inflammatory drugs and bronchodilators are effective in controlling asthma.^6^ In adults, personalized asthma treatment is targeted at patients with a type 2 (T2) high asthma phenotype. It is unclear whether this classification is feasible for children. Using readily available biomarkers, people with T2 high asthma can be identified at all ages, and different phenotypes can be depicted.^42^ The level of C4Ma3 is elevated in the phenotype of severe and aggravating allergic asthma, and C4Ma3 can be used as a new biomarker to predict the response to anti IgE treatment.^43^ IgA positive memory B cells are significantly increased in patients with asthma and small airway dysfunction, which indicates the direction for future selection of asthma prevention and treatment strategies guided by B cells.^44^

Currently, airway remodeling in asthma remains an intractable challenge. According to previous reports, lncRNAs play a significant role in regulating the proliferation and migration of ASMCs. For example, overexpression of lncRNA metastasis‐associated lung adenocarcinoma transcript 1 (Malat1) can induce proliferation and migration through miR‐150‐eIF4E/Akt signaling.^36^, ^45^ LncRNA‐maternally expressed gene 3 (MEG3) was reported to regulate Treg and Th17 homeostasis in asthmatics by targeting miR‐17 and RORγt as competitive endogenous RNA.^46^ Additionally, lncRNA plasmacytoma variant translocation 1 (PVT1) aggravates inflammation and barrier damage during asthma through the regulation of miR‐149.^47^ This evidence indicates that lncRNAs play a critical role in airway remodeling and inflammation caused by asthma. Furthermore, lncRNA ANRIL acts as a ceRNA to regulate miRNAs.^28^, ^34^, ^45^ MiR‐7‐5p is targeted directly to lncRNA ANRIL and is involved in the regulation of diseases. It has been reported that lncRNA ANRIL enhances osteogenic differentiation via regulating miR‐7‐5p, which suggests that miR‐7‐5p plays an important role in regulating bone regeneration in periodontitis.^35^ Additionally, lncRNA ANRIL is involved in T‐cell acute lymphoblastic leukemia via the miR‐7‐5p and TCF4 axis.^34^ However, the effects of lncRNA ANRIL and miR‐7‐5p on airway remodeling are unclear.

Fibroblast‐to‐myofibroblast transformation (FMT) is one of the major mechanisms of early airway remodeling.^48^ Previous studies on FMT in asthma have identified multiple precipitating factors, among which humoral factors such as cytokines, chemokines, and growth factors play important roles in phenotypic transformation. Among them, PDGF‐BB is widely used to induce ASMC proliferation and migration and aggravate airway remodeling.^35^, ^49^ Thus, in this study, PDGF‐BB‐induced HASMCs mimic airway remodeling in asthma, and the significant enhancement of HASMC proliferation and migration indicated the success of the model.^36^, ^50^ In this study, we found that lncRNA ANRIL was upregulated and miR‐7‐5p was downregulated in the serumof patients with asthma and PDGF‐BB‐induced HASMCs. In HASMCs, lncRNA ANRIL negatively regulates miR‐7‐5p. Additionally, we found that downregulation of lncRNA ANRIL inhibited the proliferation and migration of HASMCs induced by PDGF‐BB by sponging miR‐7‐5p. These results imply that lncRNA ANRIL is involved in asthma‐related airway remodeling through the regulation of miR‐7‐5p. To explore the mechanisms of action of lncRNA ANRIL in asthma, we used bioinformatics to predict the downstream targets of miR‐7‐5p. These results indicated that miR‐7‐5p has binding sites for EGR3.

EGR3 is an early transcription factor involved in the regulation of extracellular signaling.^51^ EGR3 is found to be involved in neurodevelopmental processes.^52^ For example, EGR3 regulates neuronal growth through the Reelin signaling pathway.^53^ Additionally, it is highly expressed in various tumors and is involved in tumor progression.^54^ Studies have shown that EGR3 regulates estrogen‐mediated invasion of breast cancer cells. Furthermore, it negatively regulates the cell function of T‐cells.^55^ EGR3 can also regulate pro‐inflammatory genes and directly activates IL6 and IL8 expression.^56^ However, its effects in asthma and its relationship with the lncRNA ANRIL or miR‐7‐5p have not been investigated.

The study demonstrated that EGR3 was increased in the serum of patients with asthma and PDGF‐BB‐induced HASMCs and was negatively regulated by miR‐7‐5p. We found that miR‐7‐5p inhibited ASMCs' proliferation and migration of ASMCs by suppressing EGR3, whereas the effect of miR‐7‐5p on airway remodeling was reversed by the upregulation of EGR3. These results revealed that downregulation of lncRNA ANRIL alleviates airway remodeling in asthma and that lncRNA ANRIL is a new therapeutic target for asthma.

This study is the first to elucidate the roles and potential mechanisms of lncRNA ANRIL in airway remodeling and to explore the relationship between lncRNA ANRIL, miR‐7‐5p, and EGR3 in asthma. However, the effect of lncRNA ANRIL silencing on EGR3 requires further validation. Additionally, this study was primarily based on in vitro cellular studies to investigate the roles of lncRNA ANRIL in asthma, while studies need to be further validated using animal models in vivo. We will perform these issues in the next research.

## 5 CONCLUSION

The findings of this study indicated that downregulation of lncRNA ANRIL inhibits airway remodeling by regulating the miR‐7‐5p/EGR3 signaling pathway to inhibit the proliferation and migration of ASMCs. These results revealed that the lncRNA ANRIL inhibition plays a protective role in asthma. Thus, lncRNA ANRIL may be used as a potential therapeutic target for the treatment of asthma.

## AUTHOR CONTRIBUTIONS

Liyan Wang contributed to the study design, data collection, statistical analysis, data interpretation, and manuscript preparation. Xueru Liu contributed to data collection, statistical analysis, and manuscript preparation. All authors read and approved the final manuscript.

## CONFLICT OF INTEREST STATEMENT

The authors declare no conflict of interest.

## Data Availability

The data sets used and/or analyzed during the present study are available from the corresponding author on reasonable request.
